# IgG4-Related Disease Inflamatory Pseudotumor Affecting the Sternoclavicular Joint: A Clinical Challenge

**DOI:** 10.1155/crii/6543528

**Published:** 2025-08-28

**Authors:** Orivaldo Alves Barbosa, Talita Guimarães Andrade, Sergio Saldanha Freire Simões, Andre Luis Coutinho de Araújo Macedo, Dower Frota Barroso, João Paulo Uchoa Fontenele, José Walter Correia

**Affiliations:** ^1^Department of Internal Medicine, General Hospital Dr. César Cals, Avenida do Imperador, 545, Centro, Fortaleza, Ceará, Brazil; ^2^Internal Medicine Residency Program, General Hospital Dr. César Cals, Avenida do Imperador, 545, Centro, Fortaleza, Ceará, Brazil; ^3^General Hospital Dr. César Cals, Avenida do Imperador, 545, Centro, Fortaleza, Ceará, Brazil; ^4^Department of Radiology, São Carlos Imagem, Rua Otoni Façanha de Sá, 69, Dionísio Torres, Fortaleza, Ceará, Brazil

**Keywords:** immunoglobulin G4-related disease, inflammatory pseudotumor, soft tissue neoplasms, sternoclavicular joint disorders

## Abstract

IgG4-related disease (IgG4-RD) is a rare, progressive, and immune-mediated fibroinflammatory disorder that primarily affects middle-aged men and is more prevalent in Asian populations. Although extensively studied, its pathophysiology remains incompletely understood. This case report describes a 44-year-old male presenting with multiple abscesses and progressive inflammatory symptoms, ultimately diagnosed with IgG4-RD with musculoskeletal involvement. Imaging and histopathological evaluation confirmed osteolytic lesions and significant IgG4-positive plasma cell infiltration. Soft tissue tumors in IgG4-RD are exceptionally rare, further emphasizing the uniqueness of this case. The patient showed clinical improvement with corticosteroid therapy. This case highlights the importance of considering IgG4-RD in the differential diagnosis of soft tissue and bone lesions and underscores the need for a multidisciplinary diagnostic approach.

**Summary**

• IgG4-related disease (IgG4-RD) can mimic infections or malignancies: The inflammatory pseudotumor in this case clinically and radiologically resembled abscesses and neoplastic processes, highlighting the importance of considering IgG4-RD in the differential diagnosis of soft tissue and bone lesions.
• Histopathology and immunohistochemistry are essential for diagnosis: Definitive diagnosis requires tissue biopsy demonstrating storiform fibrosis, dense lymphoplasmacytic infiltrate, and >50 IgG4 + plasma cells per HPF with an IgG4+/IgG + ratio >50%.
• Prompt corticosteroid therapy can lead to rapid clinical improvement: Early recognition and treatment with glucocorticoids are key to reversing the inflammatory process and avoiding irreversible tissue damage.

## 1. Introduction

IgG4-related disease (IgG4-RD) is a progressive, multisystemic, and immune-mediated disorder recognized for approximately two decades, yet it remains underdiagnosed. However, increasing awareness has led to a rise in reported cases. In the United States, the incidence increased from 0.78 to 1.39 per 100,000 people between 2015 and 2019 [1]. The disease predominantly affects middle-aged men and is more common in Asian populations. In Japan, the estimated incidence ranges from 0.28 to 1.08 per 100,000 people [2]. Despite significant advancements in understanding its key features and developing diagnostic tools, its pathophysiology remains incompletely elucidated.

This report presents a rare case of an IgG4-related inflammatory pseudotumor affecting the clavicular region, initially mimicking a neoplastic lesion. The unusual presentation as a tumefactive mass with osteolytic features in the sternoclavicular joint posed a significant diagnostic challenge, emphasizing the importance of considering IgG4-RD in the differential diagnosis of atypical soft tissue and bone lesions.

## 2. Case Report

The patient was illiterate and, therefore, unable to provide written consent. Verbal informed consent for the publication of this case report and any accompanying images was obtained directly from the patient, in the presence of a witness, after a full explanation of the purpose, nature, and scope of the publication.

A 44-year-old male patient with a history of psychiatric disorder and diabetes mellitus presented with abscesses on the scalp, proximal right lower limb, cervical region, and right axilla, which progressively worsened over 12 months. His condition deteriorated, with swelling and pain in the right infraclavicular region, inflammatory signs, hyporexia, and fever. He sought medical attention and underwent an ultrasound of the soft tissues, which revealed a collection. Surgical drainage was performed, and antibiotic therapy was initiated.

**Figure 1:**
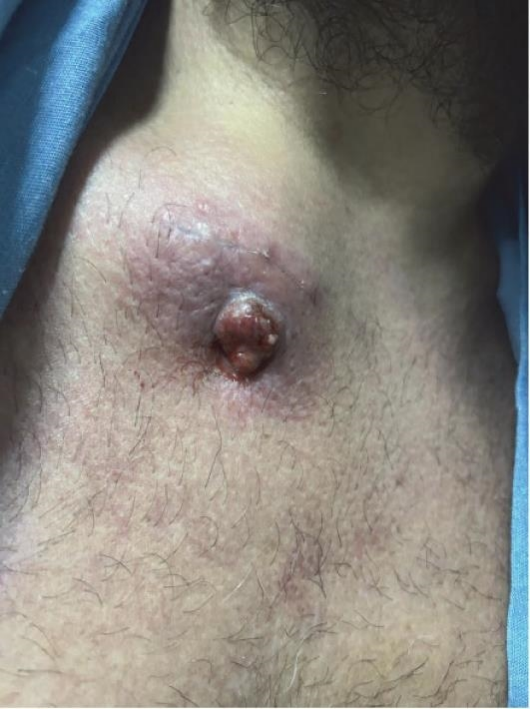
Ulcerative lesion with hyperemia and an indurated base in the right sternoclaviculr region.

Despite initial treatment, the patient continued to experience pain and erythema in the right infraclavicular region (Figure 1), prompting his return to medical care and subsequent hospitalization. A chest and neck CT scan revealed a 74 mm × 48 mm × 50 mm collection in the right infraclavicular region, along with a bone lesion at the scapulosternal joint. Initial surgical drainage and an incisional biopsy of the infraclavicular lesion revealed granulation tissue without neoplastic cells.

**Figure 2:**
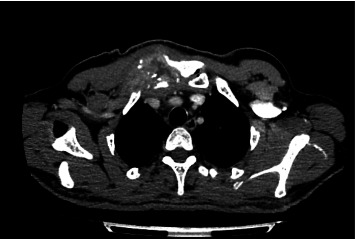
Contrast-enhanced thoracic CT scan demonstrating an expansile lytic lesion with periosteal reaction, associated with a soft tissue lesion with central necrosis and extension to the skin. The lesion involves the manubrium sterni, sternoclavicular joint, and right clavicle. Magnification: Not applicable for CT scan (displayed at standard radiological window).

A subsequent contrast-enhanced thoracic CT scan demonstrated osteolytic lesions affecting the distal third of the clavicle, the right sternoclavicular joint, and the first costal arch, along with a heterogeneous hypodense area with contrast enhancement adjacent to the sternoclavicular joint (Figure 2). A second biopsy, including a bone sample, was performed, alongside an investigation for autoimmune diseases, neoplasms, tuberculosis, and common variable immunodeficiency (Table 1).

**Figure 3:**
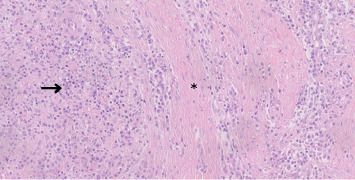
Storiform fibrosis (central area, asterisk) is characterized by swirling interwoven collagen fibers, a hallmark feature of IgG4-RD. The lymphoplasmacytic infiltrate (left side, arrow) consists of a dense cluster of inflammatory cells, including plasma cells, lymphocytes, and macrophages. Scale bar: 50 μm. Original magnification: 200×.

**Figure 4:**
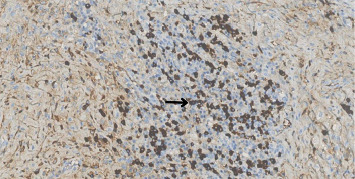
A dense aggregation of IgG4-positive plasma cells (dark brown staining) is observed, with the arrow indicating a cluster of intensely stained cells. This significant presence of IgG4 + plasma cells is a hallmark feature of IgG4-RD, highlighting their abundance in the inflammatory infiltrate. Scale bar: 50 μm. Original magnification: 200×.

Histopathological analysis of the surgical biopsy revealed a dense plasmacytic infiltrate with mild atypia (Figure 3), prompting further immunohistochemical evaluation for IgG4-RD. It is important to note that a previous incisional biopsy had provided only a small sample, which showed predominantly regenerative granulation tissue and was considered insufficient for a definitive diagnosis. In contrast, the current surgical specimen was larger and more representative. Immunohistochemistry confirmed a diagnosis consistent with IgG4-related sclerosing disease, demonstrating more than 50 IgG4+ plasma cells per high-power field (HPF) and an IgG4+/IgG+ cell ratio exceeding 50% (Figure 4). Based on these findings, prednisone therapy was initiated at a dose of 40 mg/day.

A comprehensive clinical and radiological assessment of potential organ involvement—including the pancreas, salivary glands, kidneys, and lungs—revealed no evidence of inflammatory infiltration. The patient showed progressive improvement of the lesion within 2 weeks. Given the normalization of the lesion and the resolution of the inflammatory response, a repeat biopsy was deemed unnecessary. Three months after initiating corticosteroid therapy, methotrexate was introduced at a dose of 15 mg per week to facilitate steroid tapering. At 6 months following the initial diagnosis, the patient remained free of clinical and radiological signs of inflammatory disease and was no longer receiving corticosteroids. Methotrexate maintenance therapy was planned for a total duration of 2 years.

## 3. Discussion

IgG4-RD is a recently recognized fibroinflammatory condition characterized by tumefactive lesions, a dense lymphoplasmacytic infiltrate rich in IgG4-positive plasma cells, storiform fibrosis, and elevated serum IgG4 concentrations [1]. The disease was first described in 2001 in patients with sclerosing cholangitis who exhibited elevated serum IgG4 levels [2]. Since then, several conditions have been associated with IgG4-RD, including Mikulicz's disease, autoimmune pancreatitis, hypophysitis, Riedel's thyroiditis, Küttner's tumor, interstitial pneumonia, interstitial nephritis, retroperitoneal fibrosis, inflammatory aortic aneurysm, and aortitis [3–5].

One of the frequent manifestations of IgG4-RD is inflammatory pseudotumors, which are characterized by the proliferation of inflammatory cells, including fibroblasts, myofibroblasts, lymphocytes, plasma cells, and histiocytes [6, 7]. However, IgG4-RD is rarely observed in bone and deep soft tissues [8]. In a cohort of 53 patients with IgG4-RD [9], 22 (41.5%) exhibited thoracic involvement, with mediastinal lymphadenopathy being the most common manifestation (86.4%). Other patterns included interstitial lung disease with an NSIP pattern (18.2%), pleural effusion and thickening (13.6%), a mediastinal soft tissue mass (4.5%), and only one case of inflammatory pseudotumor (4.5%). Most patients had multisystem disease beyond the thorax.

The original diagnostic criteria for IgG4-RD included three key elements: (1) organ involvement with diffuse or localized swelling; (2) elevated serum IgG4 concentrations greater than 135 mg/dL; and (3) significant plasmacytic infiltration, defined as >10 IgG4+ cells per HPF and an IgG4+/IgG+ cell ratio >40%, accompanied by fibrosis on histopathological examination [10]. This case met all these criteria.

When evaluating an indolent mass in the clavicular region suspected to be a soft tissue tumor, it is crucial to consider a broad differential diagnosis, including infectious, neoplastic, and inflammatory conditions [11]. Infectious causes include Pott's disease of the sternoclavicular joint, bacterial osteomyelitis, and fungal osteomyelitis. Among neoplastic conditions, primary bone tumors such as chondrosarcoma—a slow-growing but destructive tumor often affecting the sternoclavicular joint—should be considered, along with plasmacytoma, multiple myeloma, and metastatic disease. Inflammatory diseases can also present as clavicular masses, including sarcoidosis, IgG4-RD, and SAPHO syndrome (synovitis, acne, pustulosis, hyperostosis, osteitis), frequently associated with chronic sternoclavicular involvement and bone destruction [12].

A comprehensive approach involving clinical evaluation, imaging studies (contrast-enhanced CT or MRI), histopathological analysis, and immunohistochemistry is crucial for establishing the correct diagnosis and guiding appropriate management.

## 4. Conclusion

This case highlights the complexity of diagnosing IgG4-RD, particularly in rare sites such as the clavicular region. The confirmation of IgG4-RD through histopathology and immunohistochemistry, along with the rapid clinical response to corticosteroid therapy, reinforces the need for early recognition, and intervention to prevent irreversible organ damage. Given the diverse manifestations of IgG4-RD, a multidisciplinary approach integrating clinical evaluation, imaging, and targeted biopsy remains essential for accurate diagnosis and optimal management. Further studies are needed to better define the disease spectrum, particularly in atypical presentations, such as osseous involvement.

## Figures and Tables

**Table 1:** Results of immunoglobulin subtypes, autoantibodies, and complementary immunological tests.

Test	Result	Reference range
Immunoglobulin G1 (IgG1)	11,100 mg/L	4050–10,110 mg/L
Immunoglobulin G2 (IgG2)	4060 mg/L	1690–7860 mg/L
Immunoglobulin G3 (IgG3)	348 mg/L	110–850 mg/L
Immunoglobulin G4 (IgG4)	10,400 mg/L	30–2010 mg/L
Immunoglobulin A (IgA)	263 mg/L	85–385 mg/L
Total immunoglobulin G (IgG)	2080 mg/L	564–1765 mg/L
Immunoglobulin M (IgM)	70.4 mg/L	45–250 mg/L
Total immunoglobulin E (IgE)	617 IU/mL	<100 IU/mL
Total prostate-specific antigen (total PSA)	0.609 ng/mL	<2.5 ng/mL
Antinuclear antibody (ANA/FAN)	Nonreactive	Nonreactive
Total complement activity (CH50)	56.76 U/mL	41.68–95.06 U/mL
Anti-la (SS-B) antibody	<7.0 U/mL	<7.0 U/mL
Anti-ro (SS-A) antibody	<20.0 U	<20.0 U
Interferon-gamma release assay (IGRA)	Nonreactive	Nonreactive

## Data Availability

All data supporting the findings of this case report are included within the manuscript. No additional data are available.
